# Importance of Early Next-Generation Sequencing in Microsatellite Unstable Colon Cancer With a High Tumor Mutation Burden

**DOI:** 10.7759/cureus.22894

**Published:** 2022-03-06

**Authors:** Sethi Ashish, Moses Raj

**Affiliations:** 1 Medical Oncology, Allegheny Health Network, Pittsburgh, USA; 2 Hematology & Oncology, Allegheny Health Network, Pittsburgh, USA

**Keywords:** next-generation sequencing (ngs), msi high, metastatic colorectal cancer, polo trial, olympia trial, brca gene mutation - predominantly brca2, keynote 177, colon cancer, microsatellite instability (msi), tumor mutation burden

## Abstract

Colon cancer is one of the leading causes of cancer-related deaths. Microsatellite instability (MSI) or deficient mismatch repair proteins with a high tumor mutation burden (TMB) colon cancer are less responsive to chemotherapy. Targeted therapies based on early next-generation sequencing (NGS) in metastatic colon cancer can help significantly in overall prognosis. Here, we report a case of colon cancer that illustrated significant TMB and MSI and responded poorly to treatment due to delay in NGS testing.

## Introduction

Microsatellite instability (MSI)-high or deficient mismatch repair (dMMR) proteins have been seen in many malignancies, including the gastrointestinal and genitourinary systems [1]. Colon and endometrial cancer, in particular, have been reported to have higher MSI prevalence [2]. Roughly 12-15% of patients with colorectal cancer (CRC) and 4% of patients with metastatic colorectal cancer (mCRC) are classified as MSI-high or dMMR type [3]. Next-generation sequencing (NGS) has been widely applied in CRC screening, diagnosis, and treatment in clinical settings [4]. New treatment regimens with immune checkpoint inhibitors (ICIs) or combination therapies based on molecular profiling for appropriate therapy have the potential to improve overall survival (OS) in aggressive colon cancer.

## Case presentation

**Figure 1:**
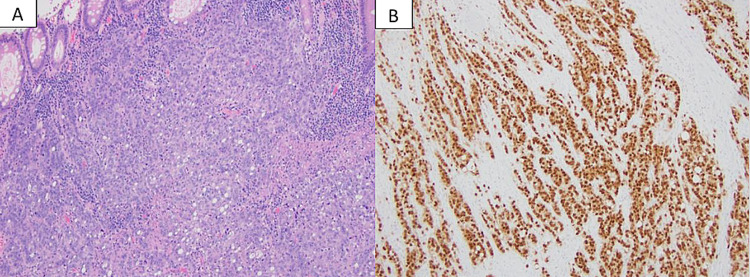
Surgical pathology: colonic adenocarcinoma. Surgical resection reveals a poorly differentiated adenocarcinoma with infiltrating lymphocytes and features of dMMR carcinoma (A, H&E stain). The tumor cells are strongly positive for CDX2 (B, immunostain), supporting a diagnosis of colonic adenocarcinoma. Permission was taken from Leo et al. [5]. dMMR: deficient mismatch repair; H&E: hematoxylin and eosin

**Figure 2:**
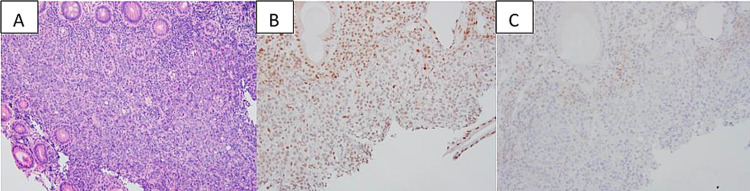
Immunohistochemical results of the biopsied colonic adenocarcinoma. Biopsy of the cecal mass reveals sheet of cancerous cells (A, H&E stain). The cells show loss of nuclear immunoreactivity for MLH1 (B, immunostain) and PMS2 (C, immunostain) in cancer. The overall features support a diagnosis of colonic adenocarcinoma with dMMR. Permission was taken from Leo et al. [5]. H&E: hematoxylin and eosin; MLH1: MutL homolog 1; PMS2: mismatch repair PMS1 homolog 2; dMMR: deficient mismatch repair

We report the case of a 73-year-old male with metastatic colon cancer who had a delay in NGS testing due to surgery and declining performance status. The patient presented in the emergency room (ER) with complaints of abdominal pain, bloating, and weight loss. He had started to feel unwell about one year ago. His symptoms were associated with fatigue and decreased appetite. The patient also reported a weight loss of 25 pounds in three weeks. Computerized tomography (CT) of his abdomen showed an ileocecal mass with small bowel obstruction (SBO). Subsequently, the patient underwent an open right colectomy with end ileostomy. Intraoperative findings revealed diffuse carcinomatosis involving the anterior abdominal wall in the pelvis and abdominal mesentery. There was also a large mass occupying the cecum and obstructing the small bowel and the appendix with mild ascites. Surgical pathology revealed stage III or T4aN2bM0 invasive poorly differentiated adenocarcinoma with mucinous and signet ring cell differentiation (Figure 1). Carcinoma was noted to invade the visceral peritoneum and appendix by direct extension. There was extensive lymphovascular invasion (LVI) and 17/19 lymph nodes were involved. Immunohistochemistry (IHC) testing showed loss of nuclear expression of MutL homolog 1 (MLH1) and mismatch repair PMS1 homolog 2 (PMS2) proteins (Figure 2). The patient had prolonged recovery time post-surgery which led to a delayed referral to the oncologist for further management. During the colorectal multidisciplinary team meeting, adjuvant chemotherapy was suggested in view of metastatic disease and diffused carcinomatosis. The plan was to complete eight cycles of a combination of folinic acid, fluorouracil, and oxaliplatin (FOLFOX) chemotherapy for over six months prior to the result of NGS. After receiving the first cycle of FOLFOX, the treatment plan was switched to pembrolizumab based on somatic NGS testing (Tables 1, 2). Molecular testing or NGS revealed a high TMB of 62.1 mutations per megabase (m/MB) and MSI (Table 3). Unfortunately, the patient only completed one cycle of pembrolizumab and died three weeks later.

**Table 1:** Genomic variants: Somatic, potentially actionable. GOF: gain of function; LOF: loss of function; BRCA2: breast cancer gene 2; PIK3CA: phosphatidylinositol-4,5-bisphosphate 3-kinase catalytic subunit alpha; KRAS: Kirsten rat sarcoma viral oncogene homolog

Somatic/Gene type	Mutation effect	Variant allele fraction: Percentage (%)
BRCA2	p.N1784fs frameshift-LOF	13.7
PIK3CA	p.R88Q missense variant (exon1)-GOF	8.3
KRAS	p.G12D missense variant (exon 2)-GOF	7.3
PIK3CA	p.EK42K missense variant (exon 9)-GOF	7.2

**Table 2:** Genomic variants: Somatic, biologically relevant. MSH6: MutS homolog 6; MSH3: MutS homolog 3; ASXL1: additional sex combs transcriptional regulator 1; CCTF-11: zinc finger protein or CCCTC-binding factor; CUL3: Cullin based E3ligase; PTCH1: protein patched homolog 1; TP53: tumor protein p53; RB1: retinoblastoma protein; FLCN: folliculin; BCOR: BCL6 corepressor; CTNNB1: catenin beta 1; CHD2: chromodomain helicase DNA binding protein 2; SETD2: SET domain containing 2, histone lysine methyltransferase; ARID2: AT-rich interaction domain 2; CUX1: Cut like homeobox 1; PPP6: protein phosphatase 6 catalytic subunit; RASA1: RAS P21 protein activator 1; FBXW7-F: Box and WD repeat domain containing 7; PPM1D: protein phosphatase, Mg^2+^/Mn^2+^ dependent 1D; CREBPP: cyclic adenosine monophosphate response element binding protein; ARID1A: AT-rich interactive domain-containing protein 1A; APC: adenomatous polyposis coli

Somatic/Gene type	Mutation effect	Variant allele fraction: Percentage (%)
*MSH6*	p.T1219I missense variant-LOF	7.7
*MSH3*	p.K383fs frameshift-LOF	7.6
*ASXL1*	p.F408fs frameshift-LOF	7.5
*CTCF*	p.H517fs frameshift-LOF	6.4
*CUL3*	p.D432fs frameshift-LOF	6.4
*PTCH1*	p.S1137fs frameshift-LOF	5.4
*TP53*	p.G245S missense variant-LOF	5.3
*RB1*	c.539+1G>A splice region variant-LOF	5.1
*FLCN*	p.H429fs frameshift-LOF	22.8
*BCOR*	p.R1164 stop gain-LOF	20.5
*CTNNB1*	p.T41A missense variant-GOF	20.1
*MSH6*	p.F1088fs frameshift-LOF	14.7
*MSH6*	p.F1088fs frameshift-LOF	12.3
*CHD2*	p.K139fs frameshift-LOF	10.4
*SETD2*	p.N1396fs frameshift-LOF	9.5
*ARID2*	p.N1778fs frameshift-LOF	9.5
*CUX1*	p.T611fs frameshift-LOF	8.9
*PPP6C*	p.F253fs frameshift-LOF	8.9
*RASA1*	p.P139fs frameshift-LOF	8.8
*FBXW7*	p.R505H missense variant-LOF	8.7
*PPM1D*	p.R572 stop gain-GOF	8.6
*TP53*	p.E204K missense variant-LOF	8.6
*CREBBP*	p.R1360 stop gain-LOF	8.3
*ARD1A*	p.D1850fs frameshift-LOF	8.2
*APC*	p.N1792fs frameshift-LOF	8.0

**Table 3:** Tumor mutation burden/MSI-high. m/MB: mutation per megabase; MSI: microsatellite instability

Tumor mutation burden	Microsatellite instability status
62.1 m/MB	High

## Discussion

Rectal bleeding or hematochezia, microcytic hypochromic anemia, altered bowel movements, abdominal pain, and weight loss are common initial clinical presentations associated with metastatic colon cancer. Lymph nodes, liver, and peritoneum are common sites of metastasis in colon cancer. NGS is a remarkable way to analyze multiple genes to identify targetable mutations. For example, a vendor like Tempus would look at 648 genes from tissue biopsy and 105 genes with liquid or plasma bases assay. For some malignancies, such as CRC, MSI, or dMMR phenotype may be a predictive factor for high TMB and respond to ICIs.

MSI-high or dMMR subtype can be detected by polymerase chain reaction (PCR) or IHC. Chemotherapies such as FOLFOX, capecitabine, bevacizumab, and ramucirumab are used as a single agent or in combination as adjuvant or palliative treatment depending upon the stage of colon cancer. Presently, ICIs such as programmed cell death protein inhibitors have been approved for MSI-high CRC first line. Phase III KEYNOTE-177 trial is a classic example of high response rates with immunotherapy compared to chemotherapy [6]. Pembrolizumab monotherapy demonstrated a statistically significant improvement in progression-free survival (PFS) compared with chemotherapy. Median PFS in the trial illustrated 16.5 months versus 8.2 months, with a hazard ratio (HR) of 0.60 and 95% confidence interval (CI) of 0.45-0.80; two-year PFS rate was 48.3% versus 33.1%. The advantage of using pembrolizumab or ICI was consistent except for those having RAS-mutated tumors (HR = 1.19, 95% CI = 0.68-2.07) [6]. Nevertheless, despite substantial clinical benefits with ICIs, 40-50% of MSI or dMMR mCRC cases manifest resistance to ICI monotherapy [6]. TMB is a useful biomarker for the consideration of immunotherapy in several cancer types revealed by NGS. It denotes the number of mutations carried by malignant cells. High TMB in cancers may be linked with environmental factors such as chronic exposure to ultraviolet light for melanoma or tobacco exposure for lung, head, and neck cancers [7].

mCRC includes biomarkers such as B-Raf, neurotrophic tyrosine receptor kinase, KRAS G12C, and human epidermal growth factor receptor 2 are actionable as well as positive and negative predictors of the overall response. As our patient harbored a high allele fraction of somatic *BRCA2 *mutation, consideration of polyadenosine diphosphate-ribose polymerase (PARP) inhibitor such as olaparib or talazoparib to prevent progression in MSI-high colon cancer could have led to a positive outcome based on the Olympia trial and the pancreas cancer olaparib ongoing (POLO) trial. The Olympia trial illustrated that one year of adjuvant olaparib, PARP inhibitors can decrease recurrence risk and prevent progression to metastatic disease in patients with high-risk early breast cancer and *BRCA1* or *2* pathogenic variants, with low-grade toxicity index [8]. The POLO trial also showed that maintenance olaparib provided a significant PFS benefit to patients with a germline *BRCA *mutation and metastatic pancreatic cancer that had not progressed with platinum-based chemotherapy [9].

## Conclusions

Early referrals to oncologists and genomic analysis can improve survival for mCRC patients. Unfortunately, our patient presented at a very late stage with declining performance status resulting in early mortality. In this patient, with timely reporting, the addition of *BRCA1* inhibitors such as PARP inhibitors with other mutations could have yielded a better outcome. Moreover, as noted in the KEYNOTE 177 trial, MSI-high status improves the likelihood of response to ICIs.
